# Infection by the hepatitis C virus in chronic renal failure patients undergoing hemodialysis in Mato Grosso state, central Brazil: a cohort study

**DOI:** 10.1186/1471-2458-7-32

**Published:** 2007-03-12

**Authors:** Marcelo AM Santos, Francisco JD Souto

**Affiliations:** 1 School of Medical Sciences, Federal University of Mato Grosso, Cuiabá, Brazil; 2 School of Pharmacy, University of Cuiabá, Cuiabá, Brazil

## Abstract

**Background:**

Hepatitis C virus (HCV) is a significant problem for patients undergoing hemodialysis therapy. This situation has never been studied in Mato Grosso state, central Brazil. This study was conducted aiming to estimate the prevalence of the anti-HCV and the incidence of seroconversion in the main metropolitan region of the state.

**Methods:**

433 patients from the six hemodialysis units were interviewed and anti-HCV was tested by a third-generation enzyme immunoassay. An open cohort of patients who tested negative for anti-HCV at the entry of the study was created and seroconversions was assessed monthly. The staff responsible for the units were interviewed to assess whether the infection control measures were being followed. Logistic and Cox regression analysis were performed in order to assess risk factor to HCV.

**Results:**

The entry on the study took place between January 2002 and June 2005. 73 out of 433 (16.9%, CI95%: 13.3–20.8) was found to be anti-HCV reactive. The multivariate analysis indicated as risk factors associated to anti-HCV the duration of the hemodialysis treatment, the number of transfusions received, and the unit of treatment. An open cohort of 360 patients who tested negative for anti-HCV was created, with a following average of 24 (± 15) months. Forty seroconversions were recorded corresponding to an incidence density of 4.6/1000 patient-months, ranges 0 to 30 among the units. Cox regression indicated the time of hemodialysis (RR = 2.2; CI95%: 1.1–4.6; p < 0.05) and the unit where treatment was performed (RR = 42.4; CI95%: 9.9–180.5; p < 0.05) as risk factors for seroconversion. The three units with highest anti-HCV prevalence and incidence were identified as those that more frequently failed to apply control measures.

**Conclusion:**

The study demonstrated high prevalence and incidence of anti-HCV in some of the hemodialysis units. Time on hemodialysis therapy was an independent factor associated to HCV. Blood transfusion was associated with anti-HCV in initial survey but was not important in incident cases. Failure of applying control meaures was more evident in units with the highest HCV prevalence and incidence. The results suggest that nosocomial transmission was the main spread factor of HCV in the studied population.

## Background

Hepatitis C Virus (HCV) infection is a common cause of chronic liver disease worldwide [1-3]. This virus is efficiently transmitted by parenteral route. As a consequence people sharing needles and syringes or submitted to blood transfusion and frequent vascular puncture especially on nosocomial environment are under increased risk. Antibodies against HCV (anti-HCV) reach high prevalence among chronic renal failure patients undergoing hemodialysis, and it is responsible for an increase in hepatic mortality and morbidity in this population [4-7].

In the past, transmission of HCV in patients undergoing hemodialysis was associated with blood transfusions, which were often necessary [8-11]. However, even with the increase in the safety of blood products and the decrease in the need for transfusions in this population, the HCV was proven to still circulate among patients undergoing hemodialysis by ways other than transfusions [12-15]. As a consequence, several prophylactic measures have been suggested to avoid infection by HCV in the hemodialysis environment, from isolating the patients carriers of HCV [15-18], to adopting a series of biosafety measures specific for hemodialysis such as preparing medications in a separated area, cleaning and disinfecting dialysis station surfaces, washing hands and changing gloves between patient contacts, and items dedicated for use only on a single patient [3,14,19,20].

International studies in hemodialysis patients have reported HCV seroprevalence rates ranging from 2.6% in Japan to 30.0% in Italy [2,21]. In Brazil, prevalence rates were reported to range from 11% to 90%, in hemodialysis units in different cities throughout the country [4,8,12,22,23].

It is important to know the prevalence and incidence of infection by HCV in local hemodialysis units so that changes can be proposed and the risks of infection among patients can be assessed. This situation has never been systematically studied in the state of Mato Grosso, in the central region of Brazil. The aim of this study was to estimate the prevalence and the rate of seroconversion to anti-HCV, and identify possible risk factors for patients in all hemodialysis units in the metropolitan area of the capital of the state of Mato Grosso.

## Methods

Data were collected on chronic renal failure patients that had been undergoing hemodialysis treatment for at least one month, at all units that provide renal facilities in the metropolitan area of Cuiabá, Mato Grosso, from January 2002 to June 2005. The great majority of patients were dependent of Brazilian Public Healthcare System.

Information on the patient and their medical records was collected, regarding the risk of transmission of HCV, such as the duration of the hemodialysis treatment, the number of transfusions received, the use of injectable drugs, the number of hemodialysis units visited, and sexual habits. The nurses responsible for the staff at the studied clinics were interviewed in order to assess whether the prevention measures recommended by the US Center for Disease Control and Prevention were being followed.

Serologic tests for detection of anti-HCV were performed monthly using third generation microparticle enzyme immunoassays (MEIA) (Axsym^® ^HCV, version 3.0, Abbott, Wiesbaden, Germany). All patients confirmed to carry anti-HCV by repeated tests on new blood samples were considered positive.

The anti-HCV results were assessed starting in January 2002, for patients that had already been undergoing hemodialysis treatment, as well as those patients who started their treatment in the subsequent months, up to June 2005. The monthly results of these tests were computed so as to create an open cohort of anti-HCV negative patients that allowed an analysis of the risk of infection by HCV during the study.

All anti-HCV positive patients were referred to hepatology service. Permission to conduct the study was obtained from the technical directors of each one of the six clinics involved. The patients who agreed to participate in the study signed consent forms stating that they were doing so freely. The protocol for the study was approved by the Committee of Ethics for Research on Human Beings of the University of Cuiabá – UNIC (protocol No. 003/04).

The data obtained were stored using the software EpiData^® ^3.0 (The EpiData Association, Odense, Denmark, 2003). A transverse analysis was performed on the results of the first laboratory assessment of anti-HCV on the participants, comparing the confirmed anti-HCV-positive patients with the group of anti-HCV-negative patients. Appropriated statistical tests were performed to compare continuous and categoric variables, with respective dispersion and confidence interval of 95% (CI95%), using the software EpiInfo 2002 (*Centers for Disease Control and Prevention*, Atlanta, US).

A logistic regression model was created to analyze the association of positive anti-HCV with the co-variables associated in the univariate analysis, at an alpha error rate of less than 0.1. For this analysis, a non-automated method was used, with the help of the software SPSS 13.0 for Windows (SPSS INC., Chicago, US, 2004).

For the analysis of the cohort of patients who initially tested negative for anti-HCV, probability curves for seroconversion over time were created using the Kaplan-Meier method, as well as Cox regression models to adjust the variables of interest in SPSS 13.0. Hazard risks (HR) were presented with their respective CI95%.

## Results

The results from 433 individuals undergoing hemodialysis in the six clinics studied were analyzed, admitted into the study from January 2002 to May 2005. The average duration of hemodialysis treatment after admission in the study was 43 months (± 36.3), varying from 1 to 204 months. The majority of the patients (84.1%) had already received transfusion of at least one unit of blood derivative product.

Of the 433 patients evaluated, 73 were anti-HCV positive, which represented prevalence in the period analyzed of 16.9% (CI95%: 13.3%–20.8%). Variation among clinics was 6.2% to 37.8%. Patient characteristics are shown on Table 1.

**Table 1:** Epidemiologic characteristics of patients undergoing hemodialysis treatment at entry of the study, per unit

	Hemodialysis Units						
	1	2	3	4	5	6	Total

Patients	116	37	58	85	72	65	433
Sex Male (%)	60.3	59.5	63.8	64.7	63.9	52.3	61
Age (m ± sd)*	48 (15)	50 (16)	49 (15)	49 (15)	49 (15)	55 (14)	50 (15)
Transfusion (%)	81.9	89.2	82.8	90.6	79.2	83.1	84.1
No. transf. (%)**							
1 – 2	36.5	36.4	54.2	33.8	45.6	47.2	41.2
3 – 6	40.6	36.4	29.2	37.7	26.3	34.0	34.9
> 6	22.9	27.3	16.7	28.6	28.1	18.9	23.9
Duration HD*** (m ± sd)	41 ± 36	60 ± 35	37 ± 34	55 ± 41	33 ± 29	39 ± 34	43 ± 36
> 1 clinic visited (%)	11.2	67.6	51.7	37.6	29.2	56.9	36.5
DST (%)	15.5	24.3	31.0	31.8	26.8	26.2	25.0
Anti-HCV	24.1	37.8	17.2	12.9	8.3	6.2	16.9
CI95 (%)	16–33	22–55	9–29	7–22	3–17	2–15	13–21

*m ± sd: mean ± standard deviation;

** No. transf: number of transfusions received;

***Duration HD: duration in months of the hemodialysis treatment.

All clinics routinely restricted anti-HCV-positive patients to machines that were kept specifically for patients with this condition. The individual dialysis devices (capillaries) were reprocessed in separate rooms.

Table 2 shows the assessed data on infection control measures for the six studied hemodialysis units. The three units with highest anti-HCV prevalence and incidence were identified as those that more frequently failed to apply control measures. For the multivariate analyses, the patients were classified into two groups of clinics: group A (4, 5, 6 units), with better adherence to control measures and group B (1, 2, 3 units), with poor adherence. Anti-HCV positivity showed an independent association with the duration of the hemodialysis treatment (p < 0.001), patients having received more than six units of blood products (p < 0.005) and belonging to one of the clinics in group B (p < 0.001) (Table 3). Risk factor classically associated with HCV transmission such as illicit drug abuse and promiscuous sexual behaviour were seldom reported by participants and did not show association with anti-HCV positivity. Previous surgical procedures were also not associate to anti-HCV.

**Table 2:** Evaluation of infection control practices of each hemodialysis units

	Units					
Control measures	Group B			Group A		

	1	2	3	4	5	6

Dedicated machines	+	+	+	+	+	+
Wear and change disposable gloves	-	-	-	+	+	+
Clean and disinfect the hemodialysis station	+	-	-	+	+	+
Individual supplies and dedicated staff	-	-	-	+	+	+
Cleaning and disinfection of equipments	-	+	-	+	+	+
Clean area to prepare medications	-	-	-	-	+	+
Prevalence (CI95%)	24 (16–33)	38 (22–55)	17 (9–29)	13 (7–22)	8 (3–17)	6 (2–15)
Incidence rates (CI95%)	7.7 (3.9–11.4)	30.5 (13.3–47.8)	13 (5–21)	0	0.6 (0–1.9)	0.6 (0–1.8)

**Table 3:** Risk factor associated with the initial prevalence of HCV seropositivity at the six hemodialysis clinics

Risk Factors	N (%)	Anti-HCV + (%)	OR* adjusted (CI95%)	p
Duration HD*				
Up to 4 years	278 (64.2)	9 (3.2)	1	-
Over 4 years	155 (35.8)	64 (41.3)	21.5 (9.7–47.6)	0.000
No. transfusions				
1 – 2	150 (41.2)	20 (13.3)	1	-
3 – 6	127 (34.9)	23 (18.1)	3.8 (0.9–15.8)	0.068
> 6	87 (23.9)	27 (31.0)	4.4 (1.0–18.6)	0.045
Clinic groups				
A (4,5,6)	222 (51.3)	21 (9.4)	1	-
B (1,2,3)	211 (48.7)	52 (24.6)	4.9 (2.5–9.6)	0.000

*OR = odds ratio. ** Duration HD: duration of hemodialysis treatment. Multivariate analysis adjusted by transfusion, age group, and gender.

The 360 patients who were anti-HCV negative in the first evaluation comprised an open cohort in which the anti-HCV results were assessed monthly. In June 2005, the patients who were still being studied and who had not shown seroconversion of anti-HCV were censored. The segment lasted in average 24 ± 15 months.

**Figure 1:**
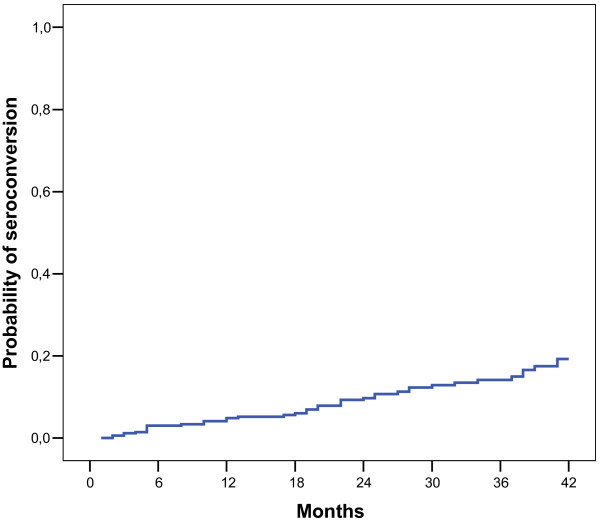
Probability curve for seroconversion to anti-HCV as a function of time, patients undergoing hemodialysis treatment.

**Figure 2:**
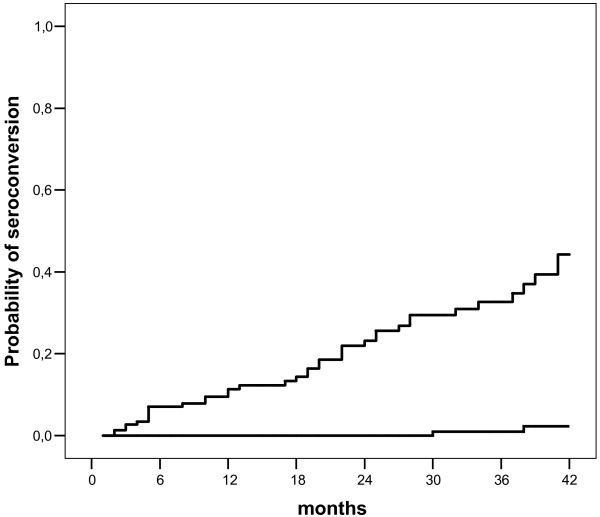
Probability curve for seroconversion to anti-HCV as a function of time, stratified by groups of clinics (A: continuous line, B: hatched line). Log Rank (Mantel-Cox): χ^2 ^= 59.3; p = 0.0001. Degree of freedom = 1.

During the segment, 40 cases of seroconversion of anti-HCV were recorded, which were irregularly distributed among the clinics. The incidence rates varied between 0 and 30.5 cases/1000 patient-months (Table 4). The probability curve for seroconversion for the set of patients is shown in Figure 1. Figure 2 shows the probability curve for seroconversion, comparing groups A and B.

**Table 4:** Density incidence of anti-HCV seroconversion per hemodialysis clinic between 2002 and 2005

Clinic	No. patients studied	Follow up average (months)	No. of events	Events/1000 patient-months (CI95%)
1	88	24	16	7.7 (3.9 – 11.4)
2	23	17	12	30.5 (13.3 – 47.8)
3	48	16	10	13 (5 – 21)
4	74	30	0	0
5	66	24	1	0.6 (0 – 1.9)
6	61	27	1	0.6 (0 – 1.8)
Total	360	24	40	4.6 (3.2 – 6.1)

The multivariate analysis of the cohort, adjusted for the variables of age group, gender, and number of transfusions, demonstrated that belonging to the clinics in group B represented a relative risk nine times higher (HR: 42.4; CI95%: 9.9%–180.5%). In addition, only the variable of having received hemodialysis treatment for more than four years at the beginning of the study showed a significant association, with a relative risk of 2.2 (CI95%: 1.1%–4.6%) (Table 5).

**Table 5:** Cox regression analysis to assess risk of anti-HCV seroconversion in patients undergoing hemodialysis therapy*

Risk Factors	N (%)	Anti-HCV + (%)	Adjusted Hazard Ratio (CI95%)	P
Time on HDT*				
Up to 4 years	269 (74.7)	25 (62.5)	1	-
> 4 years	91 (25.3)	15 (37.5)	2.2 (1.1–4.6)	0.027
Unit Groups				
A (4,5,6)	201 (55.8)	2 (5)	1	-
B (1,2,3)	159 (44.2)	38 (95)	42.4 (9.9–180.5)	0.000

*Adjusted by transfusion, number of transfusions, age group, and gender.

**HDT: hemodialysis therapy.

## Discussion

This article represents the first seroepidemiologic study on HCV spread among patients undergoing hemodialysis in the state of Mato Grosso, Brazil. The prevalence of antibodies to HCV in our sample was similar to that found in the United States, Japan, and other Brazilian states, but lower than that found by Carneiro et al. (2001) in Goiania, that is a large city also located in the central region of Brazil 900 Km apart from the region of the present study [2,4,22,23]

However, in our study, in three units (1, 2, and 3) that failed to adopt all internationally standardized infection control measures, we found higher HCV prevalence and incidence rates. Particularly in these three units items were likely shared among patients, clean and disinfection of equipments were insufficient, and a policy of glove use and handwashing was lacking. Similar findings were evidenced in other studies on hemodialysis units analysed in other large Brazilian cities [4,8,11]. Moreover, as already reported [21] higher HCV incidence rates were associated with a high burden of HCV positive patients treated in the same unit. It is likely that also understaffing [21], not studied in our report, played a role in the spread of HCV infection among patients undergoing dialysis in units with high HCV prevalence rates.

Additionally, higher incidence rates occurred in those units were a break of infection control measures was evident. This further suggests that improper practices and environmental infection control breaks, more than the blood transfusion, may represent the major factors in the HCV transmission among hemodialysis patients [12-14].

The number of transfusions received up until admission into the study was associated with higher HCV prevalence rates, but not with the frequency of new HCV seroconversions. Patients who had received transfusions before entering the study could have been exposed to HCV if they received theses transfusions before the blood banks started testing for anti-HCV and when the serologic methods were less sensitive. Moreover, as reported by several authors [24-28] in our study the duration of the hemodialysis treatment was associated with HCV acquisition, thus suggesting a nosocomial route of transmission of HCV infection.

Another interesting point raised by our study, was the lack of association between isolation of HCV infected patients, adopted in all the units, and lower HCV incidence rates. Indeed, high incidence rates were independent from isolation policies and were associated with a break in infection control measures, including lack of glove exchange between patients, handwashing, cleaning, and disinfection.

The follow up of initially anti-HCV-negative individuals revealed 40 seroconversions. It corresponds to a cumulative incidence ranging from 0 to 30.5/1000 patient-months by units. These new infections were evidently more frequent at the clinics that had a higher anti-HCV prevalence and failure in infection control measures. Blood transfusions were not more frequent among these new infected patients than anti-HCV-negative subjects. These aspects reinforces the impression that the environment, more than the blood transfusion and other risk factors, may have been the determining factor in the transmission of HCV among hemodialysis patients [15,22,27].

There was a lack of association between anti-HCV positivity and having received hemodialysis treatment in different units, a factor appointed as important by others authors, [21,22,25]. This aspect was not verified in the present study, maybe because this factor was not frequent among our sampled patients.

Our study has some limitations. First, we did not perform assays for HCV-RNA, by using polimerase chain reaction (PCR). This method permits to evidence HCV infected individuals without antibodies to HCV, a condition that involves approximately up to 5% of patients in the hemodialysis setting [29]. However, this method is still expensive to be performed as a screening test in most of developing countries. In Brazil, immunoenzymatic assays (EIA) are routinely employed in blood banks and hemodialysis units to search for HCV-infected patients. Although anti-HCV positivity by EIA does not discriminate between patients with HCV viremia and those who had HCV cleared, it is very sensitive and may occasionally identify hemodialysis patients with very low viremia not detected by PCR. Aiming to increase EIA specificity only successively positive anti-HCV patients were considered as a case in the present study. Furthermore, the positive predict value of anti-HCV by EIA increases in high prevalence settings such as hemodialysis environment. Some of the anti-HCV positive patients detected in the first evaluation could have already had the HCV cleared. Since our goal was assessing prevalence of infection instead of viremia, EIA may be likewise useful.

Second, we did not studied some likely involved in HCV in hemodialysis settings, such as having been submitted to surgical interventions on previous months [21].

In conclusion, the lesson from our study is that implementation of a isolation policy for patients with antibodies to HCV is insufficient to prevent new HCV infections when infection control measures are lacking, especially in units with a high burden of HCV infection and, likely, understaffing. In addition, isolation does not prevent superinfections by other HCV genotypes circulating the hemodialysis setting [30].

Local public health authorities were alerted about these results in order to implement a surveillance system and retraining units personnel on recommended infection control measures in hemodialysis units.

## Conclusion

High prevalence and incidence of anti-HCV were shown in some of the hemodialysis units in Mato Grosso state, Central Brazil. Time on hemodialysis therapy was an independent factor associated to HCV. Blood transfusion was associated with anti-HCV in initial survey but was not important in incident cases. These findings suggest that nosocomial transmission assumed a pivotal role for the HCV circulation occurring throughout the follow up period. Failure of applying control measures was more evident in units with the highest HCV prevalence and incidence, reinforcing that nosocomial transmission was the main spread factor of HCV in the studied population.

## Competing interests

The author(s) declare that they have no competing interests.

## Authors' contributions

MS and FS contributed to the manuscript, in the planning and design of the study, in literature search and writing of the manuscript. MS had the main responsibility in collecting data and with descriptive statistics. FS contributed to the advanced statistics analyses. All authors read and approved the final manuscript.

## Pre-publication history

The pre-publication history for this paper can be accessed here:


